# A Novel Calibration Board and Experiments for 3D LiDAR and Camera Calibration

**DOI:** 10.3390/s20041130

**Published:** 2020-02-19

**Authors:** Huaiyu Cai, Weisong Pang, Xiaodong Chen, Yi Wang, Haolin Liang

**Affiliations:** Key Laboratory of Opto-Electronics Information Technology of Ministry of Education, College of Precision Instrument and Opto-Electronics Engineering, Tianjin University, Tianjin 300072, China; hycai@tju.edu.cn (H.C.); xdchen@tju.edu.cn (X.C.); koala_wy@tju.edu.cn (Y.W.); lianghaolin1208@tju.edu.cn (H.L.)

**Keywords:** calibration, feature point, 3D LiDAR, camera, calibration board

## Abstract

Aiming at the problems of feature point calibration method of 3D light detection and ranging (LiDAR) and camera calibration that are calibration boards in various forms, incomplete information extraction methods and large calibration errors, a novel calibration board with local gradient depth information and main plane square corner information (BWDC) was designed. In addition, the "three-step fitting interpolation method" was proposed to select feature points and obtain the corresponding coordinates of feature points in the LiDAR coordinate system and camera pixel coordinate system based on BWDC. Finally, calibration experiments were carried out, and the calibration results were verified by methods such as incremental verification and reprojection error comparison. The calibration results show that using BWDC and the "three-step fitting interpolation method" can solve quite accurate coordinate transformation matrix and intrinsic and external parameters of sensors, which dynamically change within 0.2% in the repeatable experiments. The difference between the experimental value and the actual value in the incremental verification experiment is about 0.5%. The average reprojection error is 1.8312 pixels, and the value changes at different distances do not exceed 0.1 pixels, which also show that the calibration method is accurate and stable.

## 1. Introduction

Heterogeneous multi-sensor data fusion has extensive research and applications in mobile robots [1], driverless cars [2], and other fields. Compared with a single-sensor system, multi-sensor fusion systems [3] can provide richer environmental information and complete higher-level tasks such as target detection [4], autonomous location [5], and path planning [6]. The combined application of 3D light detection and ranging (LiDAR) and camera is very common. LiDAR has high resolution, strong anti-interference ability, wide detection range, and can accurately obtain the distance and angle information of the detection target [7]. The camera has a small size, low cost, and can obtain the shape and color information of the target. They can complement each other functionally. However, LiDAR and the camera are in different spatial positions in the multi-sensor system. Therefore, the calibration of the spatial coordinate system must be completed before data fusion.

The calibration of 3D LiDAR and camera is essentially the process of obtaining the mutual conversion relationship between the LiDAR coordinate system and camera pixel coordinate system. Through that, the spatial information detected by two sensors can be correlated and fused. Commonly used calibration methods can be divided into two categories: global matching optimization method and calibration tool assisted method. The global matching optimization method uses the structural similarity between the LiDAR point cloud and image data to perform global matching and optimize the calibration results. Pandey et al. [8] completed the calibration by maximizing the mutual information between the intensity of the LiDAR frame and the gray information of the image. Castorena et al. [9] used the natural alignment of depth and intensity edges in combination with a Gaussian mixture model to solve the calibration result. It is easy to analyze a global matching optimization method that is automatic, targetless, and completely data-driven. However, because of the requirement to use features such as point cloud structures and image edges, this method requires high-precision LiDAR data and is very sensitive to image distortion. This will place high demands on the hardware system. The calibration tool assisted method completes the calibration by using auxiliary tools such as a calibration board. Due to the different forms of information obtained on the auxiliary tools, this method can also be divided into two categories: vector constraint method and feature point method. The vector constraint method was originally proposed by Zhang [10]. This type of method needs to solve the intrinsic parameters of the camera [11] first. Then use the camera and LiDAR to detect calibration aids (such as a black and white checkerboard [10], an arbitrary trihedron [12], two non-coplanar triangle checkers [13], some boxes of known size [14], etc.) at the same time. The position constraint of the plane normal vectors or other selected vectors in the two coordinate systems on the auxiliary tool is used to establish the vector constraint equations and solve the external parameters. Finally, the nonlinear optimization is generally performed to refine the solution parameters. Obviously, the vector constraint method can clearly and directly solve the intrinsic parameters of the camera and the conversion parameters between the camera and LiDAR, but this method has a large calculation cost and cumbersome steps. Moreover, multi-step calibration will introduce a large cumulative error. The feature point method directly obtains the transformation relationship between LiDAR coordinate system and the camera pixel coordinate system by selecting the feature points and obtaining their coordinates in the above two systems based on the calibration board, then calculating the transformation matrix and the intrinsic and external parameters [15] by solving the calibration matrix conversion equation [16] or using method like supervised learning [17], etc. The feature point method has low calculation cost, wide applicability, and is easy to implement. In addition, the solved transformation matrix can be directly used for point cloud projection, which can effectively avoid the cumulative error generated by the multi-step calibration.

The calibration accuracy of the feature point method mainly depends on the accuracy of the extracted feature points’ coordinates. Coordinates of feature points can be acquired by infrared imager [18] or directly using a calibration board through calculation or other methods. Infrared imagers can visualize LiDAR scanning points, but the cost of the instrument is high and it must be ensured that the sensitive frequency matches the LiDAR light frequency. In view of cost and applicability, calibration boards are commonly used in related researches.

The form of the calibration boards and their corresponding calibration methods are different. Liu [19] constructed trapezoidal and rectangular deep depressions on a white flat board, and completed the single-line LiDAR and camera calibration by using the scanning points at the intersection of the plane and the depression as the feature points. However, the angle of the depth depression of the calibration board was variable which made the calibration board unstable. However, manual labeling was used to determine the pixel coordinates of the calibration board, which led to many manual errors that seriously affected the accuracy of the calibration. The work of Dong et al. [20] and Zhuang et al. [21] is a little similar. They constructed hollow circular holes in the center of the black and white checkerboard lattices, and used the centers of the circular holes as the feature points for calibration. Without obvious features in the LiDAR point cloud, the coordinates of centers could only be approximately calculated from the point cloud of the entire circular hole. Analogously, Velas et al. [22] proposed a method for extrinsic calibration. They designed a calibration board containing four circular holes, then used the circle centers and the radius of the 3D LiDAR data and 2D image to comply a coarse calibration. Finally, a fine calibration was performed by the 3D LiDAR data captured along the circle edges and the inverse distance transform of the camera image. Ha [23] used a black and white checkerboard with a triangular hole, where he took the two intersections of the scanning line and the hole as feature points, and determined the absolute position of the laser scan data on the plane by the relative positions of the feature points. Due to the lateral resolution of LiDAR and the non-coplanarity between the hole scan data and the flat scan data, the feature points cannot be obtained by fitting and intersecting, which brought many errors into the extracted feature point coordinates. Park et al. [24] used a white diamond-shaped board as the calibration board and the vertexes of the diamond-shaped board as the feature points. Then solved the coordinates of the feature points in the LiDAR coordinate system by fitting the edge points of the diamond-shaped board. White flat board can avoid extra LiDAR noise caused by texture and some special structures. However, because of the limited lateral resolution of the LiDAR, there was no guarantee that the laser could sweep to the edge of the rhombus, which affected the solution of the coordinates of the vertex of the rhombus. There are also some researchers who use light-absorbing flat boards [25] or V-shaped boards with special shapes [26] as calibration boards. Inevitably, they all introduced large calibration error limited by the design of the calibration boards and calibration methods. It can be seen from the analysis that the feature point method is widely used for 3D LiDAR and camera calibration, the form of the calibration board is arbitrary, and the calibration methods are diverse. However, due to the design of the calibration board and corresponding method, there are too many manual interventions or approximate estimates when determining the coordinates of the feature points, which makes the feature point coordinates not accurate enough and affects the accuracy of the calibration. Moreover, there is currently no reasonable method for verifying calibration results.

This article conducts the following research on the above issues. Firstly, because one of the reasons of the error introduction is the design of the calibration board, a novel calibration board with local gradient depth information and main plane square corner information (BWDC) was specially designed. The calibration board has gradient depth information, plane corner information, and position information, which is easy to experiment and adjust. Secondly, since another factor that affects the calibration accuracy is the calibration method, this paper proposed the "three-step fitting interpolation method" for selecting feature points and accurately obtaining the coordinates of the feature points in the LiDAR coordinate system and camera pixel coordinate system. Then, we discussed various conditions that should be satisfied during the calibration to reduce artificial and random errors and improve the accuracy of the calibration results. Thirdly, we designed and carried out calibration experiments and verification experiments to evaluate the calibration results.

To present the proposed method, we organize the remainder of this paper as follows. In Section 2, we describe the basic method of 3D LiDAR and camera calibration using feature points. In Section 3, we propose the design of BWDC and corresponding calibration method. Section 4 presents the experiments and analysis, followed with conclusions and future work in Section 5, and related patents in Section 6.

## 2. Basic Method of 3D LiDAR and Camera Feature Point Calibration

**Figure 1:**
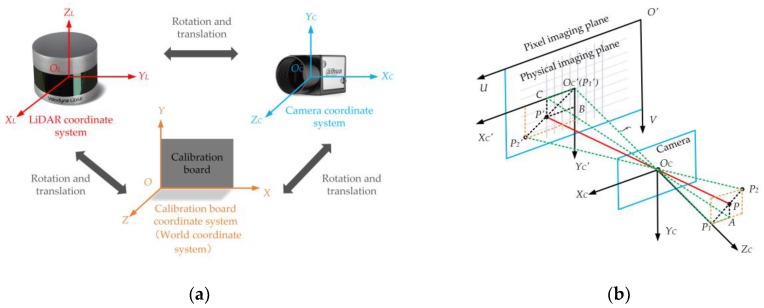
A typical schematic diagram of coordinate system conversion. (**a**) A schematic diagram of the conversion relationship between three typical coordinate systems. (**b**) An ideal pinhole camera imaging transformation model. Abbreviations: LiDAR, light detection and ranging.

The coordinate conversion relationship of a typical feature point calibration system is shown in Figure 1. Figure 1a includes the LiDAR coordinate system $O_{L}X_{L}Y_{L}Z_{L}$ (the origin $O_{L}$ is at the scan center of the LiDAR), the camera coordinate system $O_{C}X_{C}Y_{C}Z_{C}$ (the origin $O_{C}$ is at the camera’s optical center), and the calibration board coordinate system OXYZ(the origin O is at the first corner point at the bottom left). These coordinate systems can be converted to each other by rotation and translation. Figure 1b is an ideal pinhole camera imaging transformation model. An object is imaged on the image plane through the camera’s optical center pinhole $O_{C}$. Coordinate system $O_{C}^{\prime }X_{C}^{\prime }Y_{C}^{\prime }$ is the image physical coordinate system with the intersection $O_{C}^{\prime }$ of the optical axis and the image plane as the coordinate origin. Coordinate system $O^{\prime }UV$ is the image pixel coordinate system with the vertex $O^{\prime }$ of the imaging plane as the coordinate origin. The object points P,$P_{1}$, and $P_{2}$ are imaged at points $P^{\prime }$,$P_{1}^{\prime }$, and $P_{2}^{\prime }$ respectively. The camera focal length is f.

The first thing that should be clear is that the purpose of calibration is to solve the conversion relationship between the LiDAR coordinate system and the camera pixel coordinate system. We can obtain the above conversion relationship by solving the rotation vector and translation vector between the LiDAR coordinate system and the camera coordinate system, as well as the intrinsic parameters of the camera. 

The feature point calibration method is to solve the above parameters by using a sufficient number of different coordinates in two coordinate systems of the feature points. The transformation process of feature points can be divided into the following two processes: One is the linear transformation of the points between the LiDAR coordinate system and the camera coordinate system, which is the boresight alignments between camera and LiDAR and depends on the rotation vector and translation vector between the LiDAR coordinate system and the camera coordinate system. The other is the process of imaging a point in the camera coordinate system to a pixel coordinate system, which depends on the intrinsic parameters of the camera.

Firstly, according to the conversion relationship shown in Figure 1**a**, if the coordinate of a point *P* in LiDAR coordinate system is $\left(x_{L},y_{L},z_{L}\right)$ and its coordinate in camera coordinate system is $\left(x_{C},y_{C},z_{C}\right)$. Then we have

(1) $$\begin{array}{c} \left[\begin{array}{c} x_{C}\\ y_{C}\\ z_{C}\\ 1 \end{array}\right]=\left[\begin{array}{cc} R & T\\ 0 & 1 \end{array}\right]\left[\begin{array}{c} x_{L}\\ y_{L}\\ z_{L}\\ 1 \end{array}\right]\\ \begin{array}{cccc} & R=R_{x}R_{y}R_{z} & & T={\left[\begin{array}{ccc} t_{1} & t_{2} & t_{3} \end{array}\right]}^{T} \end{array}\\ R_{x}=\left[\begin{array}{ccc} 1 & 0 & 0\\ 0 & \cos\alpha & \sin\alpha \\ 0 & -\sin\alpha & \cos\alpha \end{array}\right]\begin{array}{cc} & \begin{array}{cc} R_{y}=\left[\begin{array}{ccc} \cos\beta & 0 & -\sin\beta \\ 0 & 1 & 0\\ \sin\beta & 0 & \cos\beta \end{array}\right] & R_{z}=\left[\begin{array}{ccc} \cos\gamma & \sin\gamma & 0\\ -\sin\gamma & \cos\gamma & 0\\ 0 & 0 & 1 \end{array}\right] \end{array} \end{array} \end{array},$$

where ***R*** is a 3×3 rotation matrix, which represents the angular rotation relationship between the two coordinate systems. α,β, and γ respectively represent the angles of rotation along the *x*, *y*, and *z* axes during rotating the LiDAR coordinate system to the camera coordinate system in the right-hand coordinate system. ***T*** is a 3×1 translation vector, which represents the relative position relationship between the two coordinate systems. The value is the coordinate of the origin of the LiDAR coordinate system in the camera coordinate system.

Secondly, as shown in Figure 1**b**, object point *P* is imaged by the camera to a point P′ on the pixel plane. Assume that the coordinate of point *P* in camera coordinate system is $\left(x_{C},y_{C},z_{C}\right)$ and the corresponding coordinate in image physical coordinate system is $\left(x_{C}^{\prime },y_{C}^{\prime }\right)$. Since the feature point calibration method is not particularly sensitive to camera distortion, camera distortion parameters are generally ignored in related studies. According to the similar triangle principle, we have

(2) $$x_{C}^{\prime }=f\frac{x_{C}}{z_{C}},y_{C}^{\prime }=f\frac{y_{C}}{z_{C}}.$$

According to the translation relationship between image physical coordinate system and image pixel coordinate system, the image physical coordinate $\left(x_{C}^{\prime },y_{C}^{\prime }\right)$ can be converted to the image pixel coordinate (u,v):

(3) $$u=\frac{x_{C}^{\prime }}{d_{x}}+u_{0},v=\frac{y_{C}^{\prime }}{d_{y}}+v_{0}.$$

Here, $\left(u_{0},v_{0}\right)$ is the coordinate of the pixel center of the image, that is, the pixel coordinates of the intersection $O_{C}^{\prime }$ of the optical axis of the camera and the physical imaging plane. $d_{x}$,$d_{y}$ respectively represent the pixel unit distances of the camera in the *x* direction and the *y* direction.

By Equation (2) and Equation (3), we can obtain the conversion formula of points between camera coordinate system and image pixel coordinate system:

(4) $$z_{C}\left[\begin{array}{c} u\\ v\\ 1 \end{array}\right]=\left[\begin{array}{ccc} f_{x} & 0 & u_{0}\\ 0 & f_{y} & v_{0}\\ 0 & 0 & 1 \end{array}\right]\left[\begin{array}{c} x_{C}\\ y_{C}\\ z_{C} \end{array}\right],$$

where $f_{x}=f/d_{x}$ and $f_{y}=f/d_{y}$ are the equivalent focal lengths in the *x* direction and the *y* direction respectively. $f_{x}$,$f_{y}$,$u_{0}$, and $v_{0}$ are the intrinsic parameters of the camera, which are generally provided by the camera manufacturer. $z_{c}$ can be ignored when solving equations, since homogeneous coordinates multiplied by non-zero express the same meaning.

Therefore, a total of ten parameters are required in calibration. There are three angle variables: α,β,γ in rotation vector, three position variables: $t_{1}$,$t_{2}$, $t_{3}$ in translation vector, and four intrinsic parameters of the camera:$f_{x}$,$f_{y}$,$u_{0}$,$v_{0}$.

Here, we can use the conversion equation between the LiDAR coordinate system and camera coordinate system (1), the conversion equation between camera coordinate system and pixel coordinate system (4) to derive the conversion equation between LiDAR coordinate system and image pixel coordinate system as:

(5) $$\left[\begin{array}{c} u\\ v\\ 1 \end{array}\right]=\left[\begin{array}{cccc} m_{11} & m_{12} & m_{13} & m_{14}\\ m_{21} & m_{22} & m_{23} & m_{24}\\ m_{31} & m_{32} & m_{33} & m_{34} \end{array}\right]\left[\begin{array}{c} x_{L}\\ y_{L}\\ z_{L}\\ 1 \end{array}\right]=Q\left[\begin{array}{c} x_{L}\\ y_{L}\\ z_{L}\\ 1 \end{array}\right],$$

where ***Q*** is a conversion matrix with a size of 3×4. It can be seen from the calculation process of the equation that each parameter in the matrix is a composite calculated value of several calibration intrinsic and external parameters. At this time, we transform the problem of solving the above ten calibration parameters into a problem of solving the transformation matrix ***Q***. This matrix can be calculated when the corresponding coordinates of a sufficient number of feature points are obtained. We can use this matrix to perform data space fusion directly, or solve the calibration parameters. That is, when matrix ***Q*** is solved, we can acquire twelve equations with ten unknown calibration parameters for each element in the matrix ***Q*** that is a compound operation of the calibration parameters. Through the above processes, we obtain a one-to-one correspondence between the LiDAR coordinates and the camera pixel coordinates, and complete the calibration. 

When solving the matrix ***Q*** according to the Equation (5), there is a total of twelve unknown variables, so at least four feature points which are not coplanar are needed to solve the equation, and any three of them should be non-collinear. In the feature point calibration method, we use a calibration board and a corresponding calibration method to obtain the coordinates of the feature points in two coordinate systems.

## 3. Design of the Novel Calibration Board (BWDC) and Calibration Method

### 3.1. Calibration Board Design

In order to obtain the coordinates of the feature points in the LiDAR coordinate system and pixel coordinate system respectively, the selected feature points must have obvious features in the LiDAR point cloud, and at the same time, their corresponding pixel coordinates must be easily obtained through image processing. LiDAR obtains external information by detecting distance, so we consider constructing a local gradient depth region on the calibration board, and using the points on the boundary of the region as feature points. Corner detection is simple and straightforward during image processing. Therefore, we consider establishing the relationship between these feature points and their nearby corner points, and solving their pixel coordinates by interpolating.

**Figure 2:**
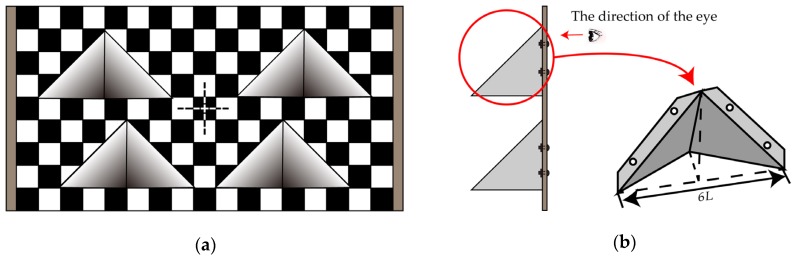
Schematic diagram of main plane square corner information (BWDC). (**a**) The elevation of BWDC. (**b**) The right elevation of BWDC and a partially enlarged depth gradient baffle structure.

Based on above-mentioned guiding ideology, the BWDC was designed as shown in Figure 2. Figure 2a is the elevation of the calibration board, and Figure 2b is the right elevation of the calibration board and a partially enlarged depth gradient baffle structure.

The main plane of BWDC is a rectangular board with a 1:2 aspect ratio and a cross mark at the geometric center. The cross mark is used as the center to generate a black and white checkerboard with the number of 9×17 and *L* set to be side length. Then, set four isosceles right-angled triangles of the same size and hollow them out, as shown in Figure 2a. In order to effectively distinguish the positions of the laser scanning lines, the interval of the triangular holes in the upper and lower rows is intentionally changed. A specially designed hollow tetrahedron baffle is connected to the back of the cavity, and its base is exactly the same size as an isosceles right triangle. After fixing, no cavity appears when looking at the calibration board. Through calculation, we can know that the height of the tetrahedron is 3*L* which is also the maximum depth of the cavity area. It can be seen that *L* is the only design parameter of the calibration board.

The calibration board parameter *L* should be selected to match the field of view and resolution of LiDAR and the camera. The minimum value can be determined by the longitudinal resolution of the LiDAR. Assuming that the longitudinal resolution range of the LiDAR is $d_{L}$, the effective distance depth of the calibration board should be greater than $3d_{L}$ to obtain at least two data points for subsequent straight-line fitting. If 80% of the gradient depth region is set as the effective region, *L* should satisfy the following relationship: $3L\cdot 20\%\geq 3d_{L}$, that is $L\geq 5d_{L}$. 

In summary, this calibration board has the following characteristics:

- There is only one parameter variable in the calibration board, that is the side length *L* of the black and white checkerboard. Therefore, the design and selection of parameter are easy and the production is convenient.
- Because the hollow structure is triangular, its different intervals between upper and lower positions and internal depth gradients make it easy to adapt sensor calibration for different parameters by adjusting the placement distance of the calibration board and selecting a suitable effective scanning line.
- The overall structure of the calibration board is symmetrical and the main plane graphics are regular, so it has the function of coarse orientation calibration. By analyzing the symmetry of the depth and height of the point cloud in the effective area and the shape of the black checkerboard after imaging, we can make a preliminary judgment on the orientation of the calibration board.

### 3.2. Calibration Method

Using the calibration board through "three-step fitting interpolation", the corresponding coordinates of the feature points in LiDAR coordinate system and camera pixel coordinate system can be calculated. Then bring the coordinates into the conversion Equation (5) and complete the calibration. The "three-step fitting interpolation method" mainly includes: selecting feature points and fitting to find their coordinates in the LiDAR coordinate system, calculating the coordinates of the feature points in calibration board coordinate system, and interpolating the feature points in camera pixel coordinates.

#### 3.2.1. Feature Points Selection and LiDAR Coordinates Calculation

**Figure 3:**
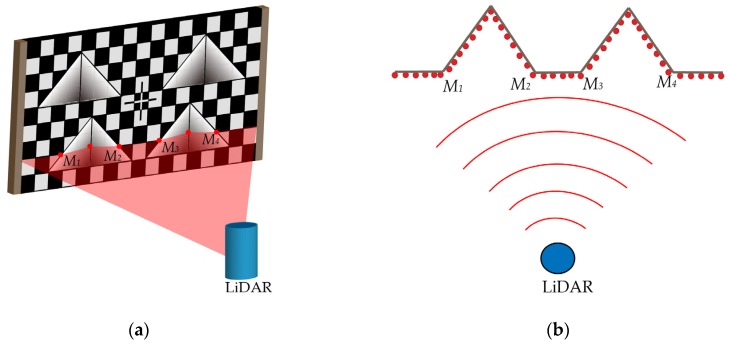
Schematic diagram of LiDAR scanning BWDC. (**a**) The stereo view of LiDAR scanning BWDC. (**b**) The horizontal cross-sectional view of LiDAR scanning BWDC.

According to the structure of the BWDC, there will be six distance abrupt points in any LiDAR scanning lines in the effective area. Select four abrupt points on the main plane as feature points and record them as *M_1_*, *M_2_*, *M_3,_* and *M_4_* from left to right, as shown in Figure 3a.

The coordinates of the feature points in LiDAR coordinate system can be obtained by fitting the scanning points of each segment to the straight line and finding the intersection of each segmented line, as shown in Figure 3b.

According to the coordinates of *M_1_*, *M_2_*, *M_3,_* and *M_4_*, the distance marked $\left|M_{1}M_{2}\right|$, $\left|M_{2}M_{3}\right|$, $\left|M_{3}M_{4}\right|$ between two adjacent feature points can be obtained. Based on this, we can judge the position of the scanning line on the calibration board using the following method.

The specific judgment method is:

- $\left|M_{1}M_{2}\right|<2L$ and $7L<\left|M_{2}M_{3}\right|\leq 9L$ indicate that the scanning line is in the second row of the grid from top to bottom;
- $2L<\left|M_{1}M_{2}\right|\leq 4L$ and $5L<\left|M_{2}M_{3}\right|\leq 7L$ indicate that the scanning line is in the third row of the grid from top to bottom;
- $4L<\left|M_{1}M_{2}\right|\leq 6L$ and $3L<\left|M_{2}M_{3}\right|\leq 5L$ indicate that the scanning line is in the fourth row of the grid from top to bottom;
- $\left|M_{1}M_{2}\right|<2L$ and $5L<\left|M_{2}M_{3}\right|\leq 7L$ indicate that the scanning line is in the sixth row of the grid from top to bottom;
- $2L<\left|M_{1}M_{2}\right|\leq 4L$ and $3L<\left|M_{2}M_{3}\right|\leq 5L$ indicate that the scanning line is in the seventh row of the grid from top to bottom;
- $4L<\left|M_{1}M_{2}\right|\leq 6L$ and $L<\left|M_{2}M_{3}\right|\leq 3L$ indicate that the scanning line is in the eighth row of the grid from top to bottom.

#### 3.2.2. Calculation of the Coordinates of the Feature Points in Calibration Board Coordinate System

**Figure 4:**
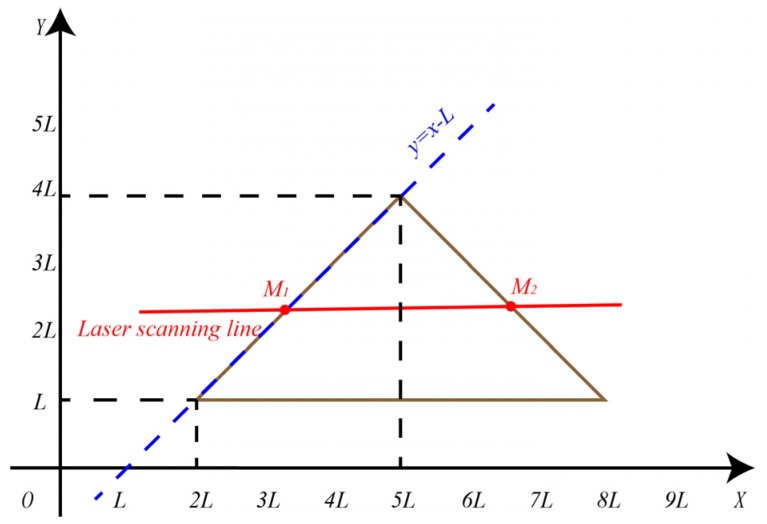
Solution of feature point coordinate on calibration board.

Referring to the calibration board coordinate system in Figure 1a and the LiDAR scan situation in Figure 3a, the line equations of the four triangular right-angle sides of the scanning line region can be calculated. As shown in Figure 4, taking the lower left triangle as an example, the abscissa of the *M_1_* point on the LiDAR scanning line in calibration board coordinate system is $x_{m1}$. Substituting the abscissa into the line equation of the right-angle side y=x−L where the *M_1_* point is located, we can obtain the coordinate of the point *M_1_* to be $\left(x_{m1},x_{m1}-L\right)$. Similarly, the coordinates of points *M_2_*, *M_3_*_,_ *M_4_* are $\left(x_{m2},-x_{m2}+9L\right)$,$\left(x_{m3},x_{m3}-8L\right)$ and $\left(x_{m4},-x_{m4}+16L\right)$.

According to the above-mentioned four feature point coordinates, distance values and collinear conditions, the coordinates of the above four points in calibration board coordinate system can be solved, as shown in Equation (6).

(6) $$\{\begin{array}{c} \sqrt{{\left(x_{m1}-x_{m2}\right)}^{2}+{\left(x_{m1}+x_{m2}-10L\right)}^{2}}=\left|M_{1}M_{2}\right|\\ \sqrt{{\left(x_{m2}-x_{m3}\right)}^{2}+{\left(-x_{m2}-x_{m3}+L\right)}^{2}}=\left|M_{2}M_{3}\right|\\ \sqrt{{\left(x_{m3}-x_{m4}\right)}^{2}+{\left(x_{m3}+x_{m4}-24L\right)}^{2}}=\left|M_{3}M_{4}\right|\\ \frac{x_{m1}-x_{m2}}{x_{m1}+x_{m2}-10L}=\frac{x_{m1}-x_{m3}}{x_{m1}-x_{m3}+7L} \end{array}.$$

After calculating the coordinates of the feature points, we can obtain the coordinates of corner points adjacent to them on the main plane of the calibration board.

#### 3.2.3. Calculation of the Coordinates of Feature Points in Camera Pixel Coordinate System

According to the similarity principle of camera imaging, their coordinates in pixel coordinate system can be solved through the relationship between the feature points and their adjacent corner points on the calibration board plane. 

As shown in Figure 1b, supposing that point P is a feature point, its adjacent corner points are $P_{1}$ and $P_{2}$, and their corresponding points after imaging are $P^{\prime }$,$P_{1}^{\prime }$,$P_{2}^{\prime }$. Then we have:

(7) $$\{\begin{array}{c} \frac{\sqrt{{\left(x_{P1}-x_{P}\right)}^{2}+{\left(y_{P1}-y_{P}\right)}^{2}}}{\sqrt{{\left(x_{P2}-x_{P}\right)}^{2}+{\left(y_{P2}-y_{P}\right)}^{2}}}=\frac{\sqrt{{\left(u_{P1}-u_{P}\right)}^{2}+{\left(v_{P1}-v_{P}\right)}^{2}}}{\sqrt{{\left(u_{P2}-u_{P}\right)}^{2}+{\left(v_{P2}-v_{P}\right)}^{2}}}\\ \frac{u_{P1}-u_{P}}{v_{P1}-v_{P}}=\frac{u_{P2}-u_{P}}{v_{P2}-v_{P}} \end{array}.$$

Here,$\left(x_{P},y_{P}\right)$,$\left(x_{P1},y_{P1}\right)$,$\left(x_{P2},y_{P2}\right)$ are the coordinates of P,$P_{1}$,$P_{2}$ in calibration board coordinate system, and $\left(u_{P},v_{P}\right)$,$\left(u_{P1},v_{P1}\right)$,$\left(u_{P2},v_{P2}\right)$ are the coordinates of $P^{\prime }$,$P_{1}^{\prime }$,$P_{2}^{\prime }$ in pixel coordinate system.

Through the above three steps, the corresponding coordinates of the selected feature points in LiDAR coordinate system and camera pixel coordinate system can be obtained. Getting enough feature point coordinates that meet the conditions and substituting it into the equation, we can obtain the correspondence between the points in two coordinate systems.

### 3.3. Calibration Process

**Figure 5:**
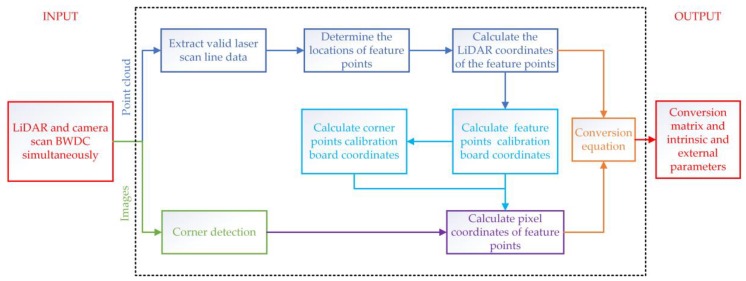
Calibration flowchart.

Figure 5 is a flowchart of 3D LiDAR and camera calibration using BWDC. Firstly, switch on the LiDAR and the camera to scan (shoot) the calibration board at the same time. Extract the LiDAR scanning line data of all valid scanning areas, and select a sufficient number of reasonable feature points. Then solve their coordinate values in LiDAR coordinate system, and calculate the distance between adjacent feature points on the same line. Secondly, judge the position of the scanning line on the calibration board according to the distance value to determine the corresponding triangle right-angle edge function that intersects the scan line. Through the above function and the collinear relationship of feature points, we can obtain the coordinates of the feature points and their adjacent corner points in the calibration board coordinates system. Thirdly, according to the imaging similarity principle, the coordinates of the feature points in pixel coordinate system can be obtained using the constraint that the collinear relationship and the distance division ratio remain unchanged among three points before and after imaging. Finally, the coordinates of all feature points in LiDAR coordinate system and camera pixel coordinate system are brought into Equation (5) to solve the transformation matrix ***Q*** and the intrinsic and external parameters.

### 3.4. Calibration Conditions

In order to ensure the accuracy and high precision of calibration, the following conditions should be noted during calibration:

- Calibration board placement distance: The closest placement distance of the calibration board is mainly limited by the camera’s field of view. When placing the calibration board, operator should ensure that the calibration board can be completely imaged. The maximum distance of the calibration board is mainly limited by the horizontal lateral resolution of LiDAR and pixel resolution of camera. That is, the following two conditions should be satisfied: (a) If the horizontal lateral resolution of the LiDAR is θ, the calibration board should be placed at a distance that meets d≤L/3θ. (b) Theoretical analysis and experimental verification show that corner point detection is more robust when a checkerboard grid is imaged with a side length of at least three pixels. Therefore, if the camera focal length is *f*, camera cell size is $L_{p}$, the calibration board should be placed at a distance that meets $d\leq f\cdot L_{l}/3L_{p}$.
- Calibration board placement angle: Excessive tilt of the calibration board will destroy the similarity of the imaging, affect the interpolation accuracy, and affect the data distribution of the point cloud in the effective area of the LiDAR. Therefore, the rough calibration needs to be performed using the self-calibration function of BWDC before the formal calibration.

## 4. Experiments and Analysis

The LiDAR model is Velodyne VLP-32C for the experiments, with a detectable range of 0–200 m, a vertical field of view angle of −25° to 15°, a horizontal field of view angle of 360°, and a horizontal lateral resolution of 0.4°. The minimum vertical lateral resolution of LiDAR is 0.33°, the detection accuracy is ±5 cm when the detection distance is less than 50 m, and the detection accuracy is ±10 cm when the detection distance is between 50 m and 200 m. The camera model is A5131CU210 from HUARAY TECHNOLOGY (Guangdong, China) with a resolution of 1280 ×1024 and a cell size of 4.8 μm × 4.8 μm. The lens model is MH0820S from HUARAY TECHNOLOGY (Guangdong, China) with the field of view of 60.8° × 42.7°, the focal length of 8 mm, and distortion of less than 0.1%. The design calibration board parameter *L* is 0.2 m, so the calibration board size is 1.8 m ×3.6 m.

**Figure 6:**
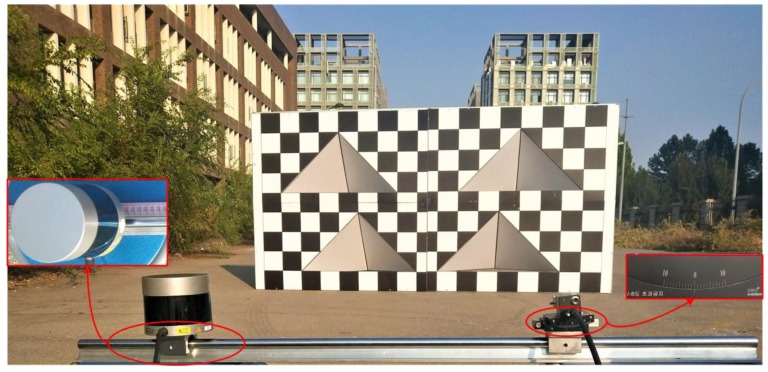
Experimental system and test scenario.

The experimental test scenario is shown in Figure 6. LiDAR and camera are respectively fixed on two sliders. The sliders can both be moved on a slide rail equipped with a ruler, wherein the camera fixed slider is provided with a readable angle rotation device.

### 4.1. Calibration Experiments

**Figure 7:**
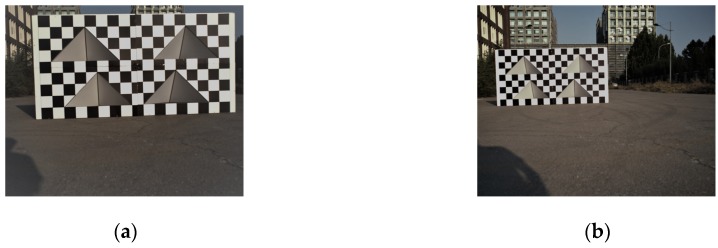

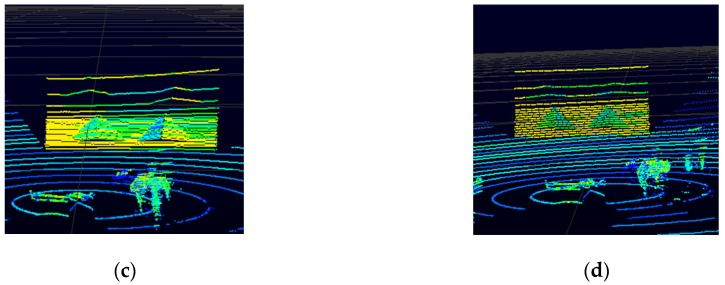
Data collected during calibration experiments. (**a**) The image data obtained by camera at 5 m. (**b**) The image data obtained by camera at 10 m. (**c**) The point cloud data obtained by LiDAR at 5 m. (**d**) The point cloud data obtained by LiDAR at 10 m.

According to the discussion in Section 2 and Section 3.4, at least four feature points not coplanar and any three of them not collinear are needed in a calibration experiment. For this reason, we placed the calibration board at the distance of 5 m and 10 m away from the sensors, and adjusted the calibration board to meet the calibration conditions. For each experimental distance, we selected three laser scanning lines in the effective scanning area. Therefore, we got twenty-four sets of feature point coordinates at the end, which is enough to complete the calibration. The data acquired by camera and LiDAR are shown in Figure 7.

Calculate the corresponding coordinates of the twenty-four feature points in the two coordinate systems. Then substitute them into Equation (5) and solve the function by the linear least square method. Calibration results are shown in Table 1.

According to the camera’s nominal value, we can obtain: $f_{x}=f_{y}=\frac{8mm}{4.8\mu m}\approx 1666.7$, $u_{0}=\frac{1280}{2}=640$,$v_{0}=\frac{1024}{2}=512$. Comparing the experimental value with the nominal value, the error is within 4%, which can initially indicate that the results of the calibration are basically accurate.

In order to verify the stability of the calibration results, without changing the relative position of the sensor and the placement distance of the calibration board, multiple calibration experiments and result calculations were performed under the same conditions. Then each calibration result was compared with the average of multiple calibration results. The rotation angle vector, translation vector, and the parameters of the camera’s parameters have a dynamic range of 0.2%. Therefore, the results of this calibration are relatively stable and not accidental data.

### 4.2. Incremental Verification Experiments

The actual relative position of LiDAR and the camera is difficult to obtain, so it is hard to quantitatively verify the calibration results by comparing experimental values with actual values. This is also the more difficult problem in the current calibration of the LiDAR and camera. For this purpose, we specially designed incremental verification experiments to indirectly verify the accuracy of the calibration results. For the experiments in Section 4.1, the relative distance and rotation angle of LiDAR and the camera were changed separately. Then we compared the changes in the calibration results with the actual values.

#### 4.2.1. Translation Incremental Verification

Keep LiDAR stationary and move the slider carrying the camera 11 cm on the guide rail. The calibration results before and after changing only the relative translation position are shown in Table 2.

Calculate the translation variation according to the two calibration results. Hence, we have

(8) $$\Delta =\sqrt{(-0.6847{)}^{2}+0.618^{2}+0.1211^{2}}-\sqrt{(-0.5743{)}^{2}+0.0442^{2}+0.11147^{2}}=0.1107m.$$

The difference between the experimental value and the nominal value is 0.0007 m, which is within the tolerance range.

#### 4.2.2. Rotation Increment Verification

Keep LiDAR stationary and rotate the angle adjustment device on the camera slider. Rotate the camera 8° counterclockwise. The calibration results before and after changing the relative rotation position are shown in Table 3.

Since the rotation direction of the camera coordinate is unknown, we use a random vector combined with an arbitrary axis rotation matrix and a multi-objective constrained programming method [27] to calculate the value. The rotation angle is 7.9542°.

The difference between the experimental value and the nominal value is 0.0458°, which is within the allowable range of the error.

Through the above two single-parameter incremental test experiments, the accuracy of this calibration method was indirectly verified.

### 4.3. Reprojection Error Evaluation

We use the more commonly known verification method in the calibration field to evaluate accuracy of this calibration method, that is the reprojection error. For the calibration experiments in Section 4.1, the pixel coordinates of the feature points after reprojection are used as experimental values here, and the pixel coordinates of feature points calculated by the "three-step fitting interpolation method" are used as theoretical values. Then, we have the reprojection error as:

(9) $$R_{e}=\frac{1}{N}\sum_{i=1}^{N}\sqrt{{\left(u_{i}-u_{i}{}^{\prime }\right)}^{2}+{\left(v_{i}-v_{i}{}^{\prime }\right)}^{2}}.$$

Here, *N* is the number of selected feature points. $\left(u_{i},v_{i}\right)$ is the theoretical coordinate. $\left(u_{i}{}^{\prime },v^{\prime }{}_{i}\right)$ is the experimental coordinate. 

In order to initially verify the accuracy of our calibration experiment, we placed the calibration board at 2, 5, 7.5, 10, 15, and 20 m respectively, then used the transformation matrix ***Q*** obtained by the experiment in Section 4.1 to solve the reprojection errors at different distances. The results are shown in Table 4.

It can be seen from the above values that the reprojection errors at different distances are all about 1.8 pixels, which can also verify the accuracy of this calibration method. Moreover, the amount of change at each distance is within 0.1 pixels. Based on this, we believe that this calibration method is basically not affected by the placement distance of the calibration board.

Finally, taking the re-projection error at various distances into consideration, we compare the results of our method with others, as shown in Table 5.

As can be seen from Table 5, the reprojection error of the method proposed in this paper is 1.8312 pixels, which is second only to the supervised learning calibration method. However, compared with our method, the supervised learning method requires a larger number of feature point samples, and the workload of the feature point acquisition process is enormous. The mutual information calibration method directly performs global optimization, and the accuracy is relatively high. However, the amount of calculation is large, and the sensor accuracy and image distortion requirements are stricter. The method adopted by Xie et al. and Jia et al. is the feature point method. It is not difficult to analyze that the accuracy is lower due to the inaccuracy of obtaining the feature point coordinates. The bi-parallel plate method is a vector constraint method. It has successively performed three calibration processes: LiDAR and car body calibration, camera and car body calibration, and camera and LiDAR calibration. The process is tedious, and the accumulated calibration errors in each process cause the overall calibration accuracy to be low. Considering the calibration accuracy, calibration workload, and convenience, the calibration method proposed in this paper has higher practical and popularization value.

## 5. Conclusions and Future Work

At present, the calibration of LiDAR and the camera using the feature point method is not perfect due to the inadequacy of the design of the calibration board and the calibration method, which results in a large error in calibration. Aiming at the above problems, this paper designs BWDC, and puts forward the "three-step fitting interpolation method" to obtain a sufficient number of accurate coordinate points of the feature points to solve the calibration matrix and the intrinsic and external parameters. The designed calibration board has both gradient depth information and corner point information. It has only one design parameter, which facilitates the control of processing errors. In addition, it has a certain self-calibration function, and is widely applicable. By applying the calibration method proposed in this paper, the corresponding coordinates of the feature points in the two coordinate systems can be directly calculated without manual labeling or approximate estimation, which greatly improves the accuracy of the feature point coordinates and further improves the calibration accuracy. It is verified by experiments that the calibration method has high accuracy, stability and accuracy. Moreover, it is not difficult to see from the general method that the proposed method is also applicable to single-line LiDAR, although developed around multi-line LiDAR.

However, the verification method proposed in this paper is still inadequate. Because the true orientation of LiDAR and the camera coordinate systems cannot be obtained, we can only use the preliminary comparison of actual and experimental values, incremental verification methods, and scanning line reprojection to verify the correctness of the calibration performance indirectly. In the future, we will consider using infrared cameras to assist verification. Other than that, this calibration method is slightly inadequate in calibration accuracy. The calibration equation is an over-constrained equation. The linear least square method used in this paper is only one of its solutions. Later, we will consider increasing the number of extracted feature points, using other optimization methods such as Gauss Newton, or training the neural networks to solve the calibration equation to further improve the calibration accuracy.

## 6. Patents

The research work in this article has applied for China invention patent. Application Number is CN201910649299.6. Publication Number is CN110322519A.

## Figures and Tables

**Table 1:** Calibration results.

Q	$\left[\begin{array}{ccc} \alpha & \beta & \gamma \end{array}{]}^{T}/\;({}^{\circ}\right)$	T/(m)	**Intrinsic Parameters**
$\left[\begin{array}{cccc} 1714.14 & 645.5 & -116.45 & -1099.39\\ -84.98 & 440.54 & -1743.2 & 170.85\\ -0.0049 & 0.9986 & -0.0526 & 0.1212 \end{array}\right]$	$\left[\begin{array}{c} -93.0318\\ 2.7745\\ 0.1203 \end{array}\right]$	$\left[\begin{array}{c} -0.6847\\ 0.0618\\ 0.1211 \end{array}\right]$	$\begin{array}{c} f_{x}=1719.3\\ f_{y}=1719.6\\ u_{0}=642.29\\ v_{0}=532.01 \end{array}$

**Table 2:** Translation incremental verification results.

Measurement	Before Translation	After Translation
T/ (m)	$\left[\begin{array}{c} -0.6847\\ 0.0618\\ 0.1211 \end{array}\right]$	$\left[\begin{array}{c} -0.5743\\ 0.0442\\ 0.1147 \end{array}\right]$

**Table 3:** Rotation incremental verification results.

Measurement	Before Rotation	After Rotation
$[\begin{array}{ccc} \alpha & \beta & \gamma \end{array}$]^T^/ (°)	$\left[\begin{array}{c} -93.0318\\ 2.7745\\ 0.1203 \end{array}\right]$	$\left[\begin{array}{c} -92.5492\\ -4.9428\\ 0.684 \end{array}\right]$

**Table 4:** Reprojection errors at different distance.

Distance/(m)	Reprojection Errors/(pixel)
2.5	1.8714
5	1.8508
7.5	1.7935
10	1.8494
15	1.7859
20	1.8336

**Table 5:** Reprojection errors of different methods.

Calibration Method	Reprojection Errors/(pixel)
Proposed method	1.8312
Supervised learning calibration [17]	0.8062
Mutual Information calibration [8]	2.8018
Xie [28] proposed method	2.9700
Trapezoid checkerboard calibration [15]	3.5691
Double parallel plate calibration [29]	4.9651
